# Degrowth – Taking Stock and Reviewing an Emerging Academic Paradigm

**DOI:** 10.1016/j.ecolecon.2017.01.014

**Published:** 2017-07

**Authors:** Martin Weiss, Claudio Cattaneo

**Affiliations:** a European Commission – Joint Research Centre, Directorate C – Energy, Transport and Climate, Sustainable Transport Unit, via Fermi 2749, TP 441, 21027 Ispra, Italy; b Autonomous University of Barcelona, Barcelona Institute of Regional and Metropolitan Studies, 08193 Bellaterra, Catalonia, Spain

**Keywords:** Degrowth, Economic growth, Sustainable development, Steady-state economy

## Abstract

Degrowth has evolved within a decade from an activist movement into a multi-disciplinary academic paradigm. However, an overview taking stock of the peer-refereed degrowth literature is yet missing. Here, we review 91 articles that were published between 2006 and 2015. We find that the academic degrowth discourse occupies a small but expanding niche at the intersection of social and applied environmental sciences. The discourse is shaped by authors from high-income, mainly Mediterranean, countries. Until 2012, articles largely constitute conceptual essays endorsed by normative claims. More recently, degrowth has branched out into modelling, empirical assessments, and the study of concrete implementations. Authors tend to agree in that economic growth cannot be sustained *ad infinitum* on a resource constraint planet and that degrowth requires far reaching societal change. Whether degrowth should be considered as a collectively consented choice or an environmentally-imposed inevitability constitutes a major debate among degrowth thinkers. We argue that the academic discourse could benefit from rigid hypotheses testing through input-output modelling, material flow analysis, life-cycle assessments, or social surveys. By analyzing the potentials for non-market value creation and identifying concrete well-being benefits, the degrowth discourse could receive wider public support and contribute to a paradigmatic change in the social sciences.

## 1 Introduction

The 2008 financial crisis has spurred research on alternative development trajectories for the global economy. Among the diverse streams of thought, degrowth has emerged as a radical call for a voluntary and equitable downscaling of the economy towards a sustainable, just, and participatory steady-state society (R&D, 2010, Schneider et al., 2010, Kallis, 2011). As a political slogan with theoretical and practical implications (Latouche, 2010), degrowth postulates that indefinite economic growth on a finite planet is impossible; facilitating growth as the overarching aim of socio-economic policy will eventually lead to involuntary economic decline with far-reaching social and political consequences.

In less than a decade, degrowth has evolved from an activist movement into a vibrant multi-disciplinary academic field, grounding in Georgescu-Roegen's (1971) thermodynamic analysis of the economy, Meadows' et al. (1972) limits to growth, and Daly, 1973, Daly, 1997 work on the steady-state economy. Degrowth resonates the anti-utilitarian ideas of Ghandi, Illich, Schumacher, and Latouche (see, e.g., Latouche, 2010, Demaria et al., 2013, Muraca, 2013), draws from anthropology, sociology, and philosophy, and links to inter-disciplinary research in ecological economics and industrial ecology (Martínez-Alier et al., 2010).

The number of peer-refereed articles on degrowth has been growing steadily since 2006. By mid-2016, six dedicated conferences had been organized, seven special issues were published, and an new special issue on degrowth and technology was already in preparation. Yet, a review of the peer-refereed literature that takes stock of the academic degrowth discourse and identifies its magnitude, trends, and unresolved research questions is yet missing. Here, we attempt such a review with the aim to (i) structure the degrowth discourse, (ii) identify areas for future research, and ultimately (iii) help devising implementable degrowth solutions.

The article continues with an explanation of our research method (Section 2) and an overview of key statistics characterizing the degrowth discourse (Section 3). We sketch important topics of the peer-refereed literature in Section 4 and identify knowledge gaps to be addressed as part of a more comprehensive research program in Section 5. The article finishes with a discussion and conclusions in 6, 7.

## 2 Materials and Methods

Our review is based on a web-search for peer-refereed journal articles in the online data base ‘Scopus’. We include research articles, comments, and editorials that contain the words ‘degrowth’ or ‘de-growth’ in their title and were published in the English language before 31 December 2015. This approach yields a timely overview of the academic degrowth literature but is subject to four limitations:

• Potentially relevant articles that do not explicitly mention ‘degrowth’ in their title are excluded (e.g., Daly, 2010, Mauerhofer, 2013b, Alexander, 2013b, Knight et al., 2013, Antal, 2014, Martínez-Alier et al., 2014, Fitzgerald et al., 2015) even if these are published as a part of a special issue on degrowth (i.e., the following 11 articles: Alcott (2010), D'Alessandro et al. (2010), Hamilton (2010), Hueting (2010), Matthey (2010), Spangenberg (2010), van den Bergh (2010), Johanisova and Wolf (2012), Latouche (2012), Tammilehto (2012), Dobson (2013)^1^).
• Research published in languages other than English is excluded (e.g., Bonaiuti, 2013).
• Contributions to the five global conferences on degrowth are excluded, if these have not been published as peer-refereed journal articles.
• Monographs (e.g., Daly, 1973, Daly, 1997, Jackson, 2009) are excluded as well as the non-peer-refereed ‘gray’ literature on degrowth.

The first limitation is justified by the need to set boundaries for our review that prevent discussions about the inclusion versus exclusion of publications while at the same time rendering the research feasible within the resources available to us. The second limitation is born out of practical constraints but could indeed be justified by the observation that English constitutes the *Lingua franca* of the global research community. The third and fourth limitations reflect our concerns about the scientific relevance of publications that are not peer-refereed. Moreover, the exclusion of conference contributions can be justified because selected research presented at the various degrowth conferences has been also published as peer-refereed articles in special issues and is thus included in our review (see Tables S1 in the Supplementary Material).

Nevertheless, we acknowledge that the academic degrowth discourse may have drawn fundamentally from publications that are excluded from this review. Jackson and Victor (2015a), for example, find that declining growth rates may not inevitably raise social inequality. This observation is relevant for the degrowth discourse but not further discussed here. We would therefore argue that the results and conclusions presented in this article are valid, strictly speaking, only for the literature included in our review. Yet, our insights could be considered indicative of the major trends and open research questions of the academic degrowth discourse in general.

To minimize errors in the interpretation of the reviewed articles, we have shared with the corresponding authors, as far as possible, our interpretation of their work. For 60 out of the 91 reviewed articles, we have received a positive response confirming our understanding of the presented research.

We complement our review by a Google search to elicit the magnitude and popularity of the degrowth discourse compared to the more general debate on environmental sustainability and economic growth. The outcome of this search is presented next.

## 3 Key Statistics of the Degrowth Discourse

**Fig. 1:**
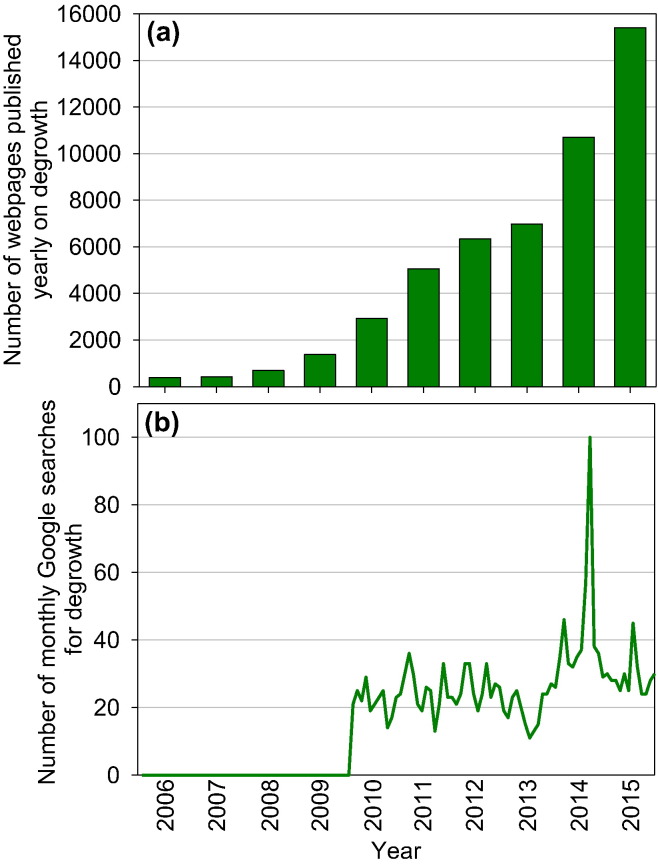
Indicative number of webpages published yearly on ‘degrowth’ (a) and number of monthly Google searches for degrowth (b). Fig. 1a only considers webpages with a publication date (n = 50,300), accounting for a quarter of the total number of webpages (253,000) listed by Google in response to the search term ‘degrowth’.

By 1 May 2016, the internet search engine Google lists 253,000 web pages in response to the search term ‘degrowth’. This number is small compared to the 101 million and 114 million pages listed in response to the terms ‘climate change’ and ‘sustainability’ and the 46.5 million webpages listed on ‘economic growth’. The term ‘post growth’ (580,000 webpages) appears to be more popular than degrowth.^2^ Still, the number of webpages on degrowth has been increasing by a factor of 20 since 2006, showing an average annual growth rate of some 150% (Fig. 1a). The number of dedicated Google searches for ‘degrowth’ are fluctuating at around 27 ± 12 per month with a pronounced peak in early 2014, presumably related to the degrowth conference in Leipzig, Germany (Fig. 1b).

**Fig. 2:**
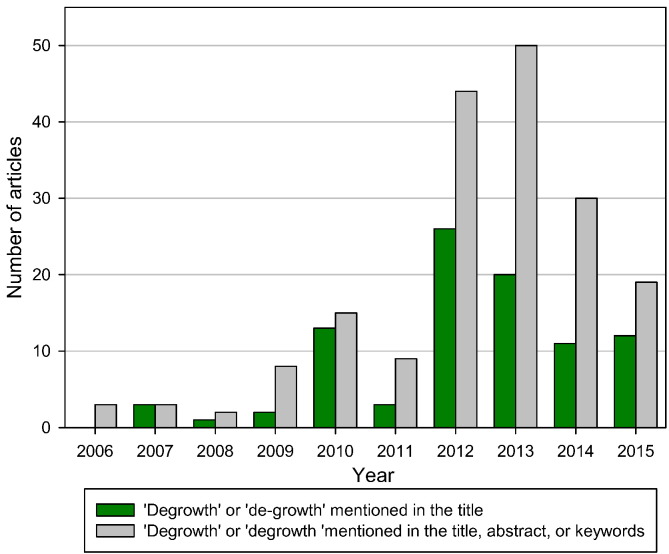
Number of peer-refereed articles explicitly referring to degrowth; this review considers only peer-refereed articles that contain the terms ‘degrowth’ or ‘de-growth’ in their title.

The relatively low but steadily increasing popularity of degrowth is also reflected by the growing number of peer-refereed articles published yearly (Fig. 2). The first articles referring to ‘degrowth’ in their title appeared in the English academic literature around the year 2006.

By 31 December 2015, 91 articles had been published (Tables A1 in the Appendix; Table S1 in the Supplementary Material). These were written by 108 authors and published in 23 journals. Twenty articles were published by the Journal of Cleaner Production, 18 by Ecological Economics, 10 by Futures, and 8 each by Environmental Values and Capitalism Nature Socialism. A first special issue on degrowth was published in 2010 by the Journal of Cleaner Production. Since then, seven special issues comprising a total of articles 53 (including editorials) have been dedicated to degrowth; an eighth special issue on degrowth and technology is to be published in fall 2016 (Kerschner et al., 2015).

**Fig. 3:**
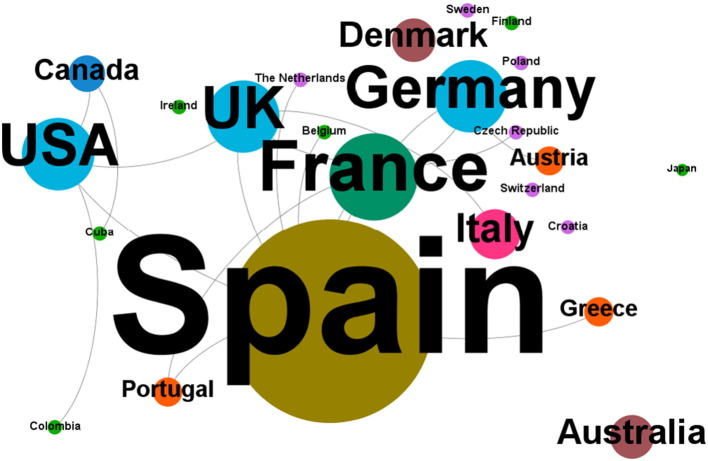
The global research arena on degrowth; the size of dots depicts the frequency at which authors affiliated in a country have published articles with the word ‘degrowth’ or ‘de-growth’ in the title; lines depict co-authorships.

**Fig. 4:**
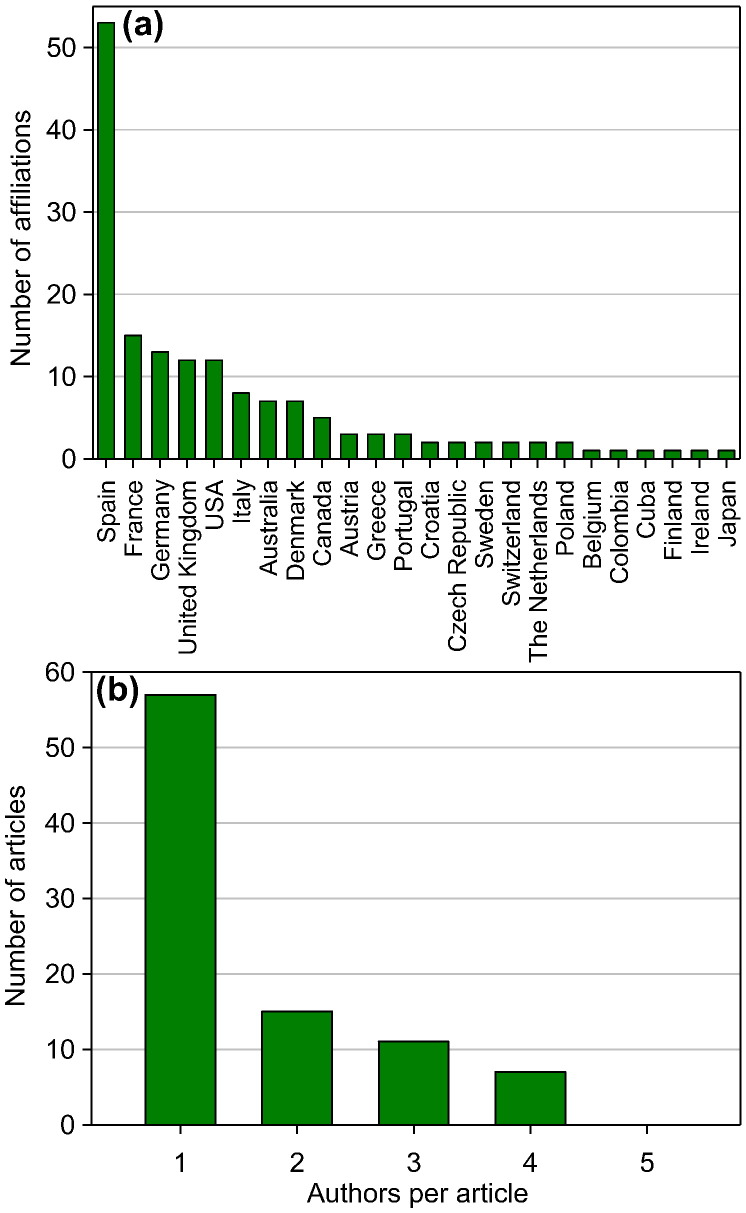
Number of author's affiliations per country (a) and number of authors per article (b). The number of author's affiliations includes cases where authors are affiliated with multiple institutions. The total number of author's affiliations does therefore not match the total number of authors that have been publishing on degrowth. We exclude from this analysis R&D (2010) that constitutes the collaborative result of the workshop “Toward a Declaration on Degrowth”, held at the first degrowth conference in Paris on 18–19 April 2008.

Authors from around the globe contribute to the academic degrowth discourse; yet the majority of articles originate from Europe, with a clear dominance of contributions from Spain (Fig. 3, Fig. 4a). This observation supports the hypothesis of Romano (2012) who suggests that the socio-economic conditions of the capitalist periphery in Mediterranean Europe may be suitable for developing and implementing degrowth.

With the exception of Boillat et al. (2012) and Escobar (2015), none of the articles were written by authors affiliated with institutions in emerging or low-income countries. The overwhelming majority of articles is written by a single author (Fig. 3, Fig. 4b), pointing to a close link of the degrowth discourse to social sciences where single-author publications are more common than in natural sciences and engineering. Moreover, co-author collaborations often remain within the same institution, suggesting a lack of international and even global exchange of views that could, however, diversify and advance the academic degrowth discourse.

When expanding our search to peer-refereed publications that refer in their title, abstract, or keywords to degrowth, we identify a total of 183 articles (Fig. 2). This observations suggest that our review indeed only addresses a part of the relevant degrowth literature.

## 4 Characterizing the Academic Degrowth Discourse

**Fig. 5:**
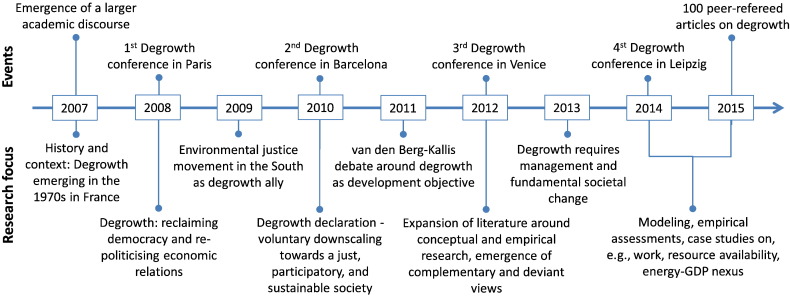
Stylized evolution of the academic degrowth discourse.

The academic degrowth discourse has emerged from the French cultural critique of the growth imaginary (Latouche, 2005) and from environmental and social activism (Demaria et al., 2013, Infante-Amate and González de Molina, 2013). It draws from the concrete experience of voluntary simplicity in co-housing communities (Lietaert, 2010), squatting (Cattaneo and Gavaldà, 2010), and neo-ruralism (Martínez-Alier, 2012). The historical ties of the degrowth discourse become apparent in two features. First, the majority of, mainly earlier, articles discusses history, context, concepts, and the motivation for degrowth in the form of structured essays that reflect the communication practice of the social sciences. Only 17 out of 91 articles separate introduction, methods, results, and discussion as it is typically done in the natural sciences. Second, one third of the reviewed articles contain normative claims that are inaccessible to rigid scientific testing, often adhering to a vision that wants to reclaim democracy and re-politicize economic relations. Grounding in the conceptual work published until 2012, research on degrowth has been recently branching out into more formal economics, material and energy flow accounting, and empirical case studies (Fig. 5; Table S2 in the Supplementary Material).

The discourse reflects, schematically speaking, two views. The first one, expressed in more than 80% of the articles, considers degrowth as an *ex-ante* policy objective, anticipating bio-physical constraints, and emphasizing the virtue of voluntary frugality and simplicity (R&D, 2010). The second one, expressed in some 20% of the articles, considers degrowth foremost as an inevitable *ex-post* socio-economic management challenge imposed by ecological or social limits to growth (e.g., Sorman and Giampietro, 2013).

The attitudes about degrowth include:

• unanimous support (e.g., Kallis, 2011, Schneider et al., 2010, Kallis and March, 2015)
• neutrality that (i) takes a positive stance to investigate degrowth scenarios (e.g., Cattaneo and Gavaldà, 2010, Xue, 2014) or (ii) uses degrowth as frame for empirical analysis (e.g., Infante-Amate and González de Molina, 2013, Kalimeris et al., 2014)
• skepticism and rejection.

About 20% of articles fall into the last category, including opposition on economic grounds (e.g., Tokic, 2012) and for practical reasons (van den Bergh, 2011, Schwartzman, 2012, Saed, 2012). Radical anarchist thinkers as Fotopoulos, 2007, Fotopoulos, 2010a, Fotopoulos, 2010b and Trainer, 2012, Trainer, 2014 consider degrowth as insufficient to address contemporary sustainability challenges; skeptics who oppose the paradigmatic degrowth vision include Romano (2012) and Sorman and Giampietro (2013).

About two thirds of the reviewed articles address matters of universal spatial and temporal validity; more than a third of all articles focuses on conceptual, philosophical, social or political aspects of degrowth. Among the articles engaging in a multi-dimensional conceptual discussion, the conceptualization of degrowth by Schneider et al., 2010, Schneider et al., 2011, the discussion of degrowth and democracy by Ott (2012), and the controversy between van den Bergh (2011) and Kallis (2011) help clarifying the scope and challenges of the degrowth proposal.

Out of the 91 articles, 18 present empirical analyses, 18 address case studies, and 6 engage in formal modelling. Six studies specifically address the emergence and historical context of degrowth (e.g., Fournier, 2008, Sippel, 2009, Muraca, 2013). Levallois (2010) presents a historic account of the collaboration between Georgescu-Roegen and the Club of Rome. Twelve articles address economic aspects of degrowth, e.g., through a description of the functional principles of the globalized market economy that tends to define a narrow space for activity in favor of increased efficiency, consumption, and private investment in man-made capital (e.g., Douthwaite, 2012, van Griethuysen, 2010, van Griethuysen, 2012).

Energy and resource use has received the attention of seven articles. Specific aspects of energy supply and demand are addressed by Alexander (2012b), Infante-Amate and González de Molina (2013), Sorman and Giampietro (2013), and Kalimeris et al. (2014). The academic degrowth discourse has been expanding into formal modelling (e.g., Bilancini and D'Alessandro, 2012, Andreoni and Galmarini, 2014, Heikkinen, 2015) and the analysis of household consumption, food and agriculture, and health (Fig. 5). Technology, has received little attention but when addressed, it is controversially discussed and often seen critically as cause rather than remedy of persisting sustainability shortfalls (Trainer, 2010, Trainer, 2014, Romano, 2012, Pueyo, 2014).

The relationship between degrowth, democracy, and work may deserve special attention. The link between degrowth and democracy is broadly and controversially discussed among authors, reflected by the quest to deepen participatory democracy as an intrinsic element of the degrowth proposal (R&D, 2010). At the same time, however, degrowth faces the paradox that democracy has historically been demanded as a *bourgeois* condition for unconstraint individual freedom and economic growth (Deriu, 2012). The issue of work has been addressed from two angles, the reduction of working time as a concrete measure to implement degrowth (e.g., van den Bergh, 2011) and the dichotomy between paid and unpaid work (D'Alisa and Cattaneo, 2013, Nierling, 2012, Andreoni and Galmarini, 2014). The anecdotal characterization of articles thus far is complemented by a detailed overview of the research presented in each individual article (Table S2 in the Supplementary Material).

## 5 Towards a Research Program on Degrowth

### 5.1 Identifying Knowledge Gaps for Future Research

From our review, we identify five cross-cutting and non-complementary domains for future research: (i) the normative foundation of degrowth, (ii) formal modelling, (iii) empirical assessments, (iv) engineering and technological innovation, and (v) the implementation of degrowth. In this section, we sketch out selected knowledge gaps in these domains before we derive in Section 5.2 research hypotheses and the contours of a research program.

#### 5.1.1 Clarifying the Normative Foundations of Degrowth

As an ‘activist-led science’ (Martínez-Alier et al., 2011, Demaria et al., 2013), degrowth has opened a new utopian imaginary (Kallis and March, 2015) based on normative claims. Yet, verifiable hypotheses scrutinizing these claims are often not developed and rigidly tested. This observation does not dismiss the normative foundations of degrowth nor do we expect that any normative claim can be put under scientific scrutiny. However, a positive rather than normative approach to degrowth can make the academic discourse more credible. The following, in part provocative, questions could serve as a reference:

• Are growth policies to be dismissed as unsustainable or could they help maintaining long-term infinitesimal growth, expanding the economy asymptotically towards the planetary boundaries (for example through further commodification of human relations or more efficient exploitation of natural resources)? If so, how do such policies impact the well-being of individuals, the functioning of societal institutions, and the environment at the various spatial and temporal scales?
• Is the globalized market economy to be dismissed as inherently unjust (Trainer, 2010, Trainer, 2014) or is there scope to redistribute welfare gains to the losers of globalization? Through which mechanisms can redistributive policies be strengthened at national and international level?
• If degrowth is to be pursued as a choice (Schneider et al., 2010) of voluntary simplicity (Alexander, 2013a), do practitioners fare better relative to control groups?

The analyses on the public perception of degrowth by Ančić and Domazet (2015) and on rurban squats as grass-root initiatives by Cattaneo and Gavaldà (2010) provide first insights into the latter question. In fact, neither proponents (such as Heikkinen, 2015) nor critics (such as Romano, 2012) have tested how fast and under which conditions grass-root initiatives can help redefine social norms in a given cultural and economic context.

Likewise, the expectation that environmentally-forced degrowth will mark a turning point in human development (Quilley, 2013) could be scrutinized through formal economic modelling or empirical assessments of the macro- and micro-economic links between resource use, pollution, and economic activity.^3^ Studies on the market potentials and sustainability effects of high value-added products (e.g., Harasym and Podeszwa, 2015) provide just one angle to address this topic.

Moreover, case studies on the societal and environmental implications of recessions can serve as test cases to understand the effectiveness of proposed degrowth strategies. The findings of Borowy (2013), Canavan (2013), and De Vogli and Owusu (2015) may not be generalized but provide an important starting point for evaluating policy interventions in face of environmentally-imposed degrowth.

#### 5.1.2 Formal Modelling

Formal economic modelling has been criticized for its spurious accuracy caused by the omission of detail and the application of rigid mathematical formulations to implausible assumptions (e.g., Daly and Farley, 2011, Andreoni and Galmarini, 2014). Therefore, the academic degrowth discourse had long abstained from employing formal modelling. Recently, however, modelling has made its way into the degrowth research (e.g., Bilancini and D'Alessandro, 2012, Andreoni and Galmarini, 2014) and proved useful to understand degrowth impacts in the environmental, economic, and societal domains. Cross-benefits could materialize from the emerging field of ecological macro-economics, including ecological econophysics (see Pueyo, 2014) that often makes use of formal models (e.g., Jackson and Victor, 2015a, Jackson and Victor, 2015b, Jackson et al., 2016, Bernardo and D'Alessandro, 2014). More specifically, formal modelling can be useful to address two, largely disregarded, aspects of degrowth: (i) the demonstration of well-being benefits^4^ and (ii) the assessment of the functional relationship between degrowth objectives at various temporal and spatial scales.

#### 5.1.3 Empirical Assessments

Most of the reviewed articles present data to furnish arguments; however, comprehensive empirical analyses of the degrowth proposal are still scarce. Focusing on the environment-economy nexus, the degrowth discourse could benefit from physical input-output analysis, material flow accounting, and life-cycle assessments. The large literature body on the environmental Kuznets-curve hypothesis is often anecdotally referenced but only recently have assessments scrutinized the hypothesis with explicit reference to degrowth objectives (e.g., Mauerhofer, 2013a, Xue, 2015, Ward et al., 2016). Analyses such as that of Kalimeris et al. (2014) on the GDP-energy link can clarify the relationship between selected economic activities and their environmental impacts.

The topic of resource extraction could receive more attention. The assessment of Exner et al. (2014) on copper reserves, in-use stocks, and substitution potentials could be expanded in a scenario analysis to other renewable and non-renewable resources, focusing also on the security of supply and resource equity at various spatial and temporal scales. Economic complexity suggests that such research may take at best a system's approach to capture both the direct and indirect impacts of resource use.

Taking a macro-economic perspective, the set of biophysical and social indicators used by O'Neill (2015) to assess the degree to which national economies are approaching a steady-state could be expanded by considering impacts on health, environment, and natural resources such as human toxicity, eutrophication, and land use that are typically quantified in the context of life cycle assessment (see JRC, 2011).

Empirical assessments are specifically suited to elicit the unintended environmental consequences of degrowth objectives (see Xue (2014). Consumption rebounds resulting from voluntary frugality have been discussed (e.g., Alcott, 2005, Alcott, 2008) but not quantified.

Empirical assessments of degrowth in a social context could start out with surveys mapping the public perception, thereby complementing and expanding the analyses conducted by Ančić and Domazet (2015) and Drews and van den Bergh (2016). Voluntary frugality has been both advocated (Schneider et al., 2010, Schneider et al., 2011) and criticized (Romano, 2012) as a concrete degrowth implementation. Yet, it remains unclear whether and under which conditions frugality and voluntary simplicity appeal to a larger part of the population in affluent countries^5^ and how such behavior can change social norms. Clarifying these points, e.g., through analyzing data from social media could help optimizing communication strategies in support of degrowth.

Questions of justice in a degrowth society are still open for exploration (Muraca, 2012). A detailed inquiry into the societal costs of the status quo (Lorek and Fuchs, 2013) and those of plausible degrowth scenarios could help putting the expected societal effects of degrowth into perspective. The analysis of the economic crisis in Cuba (Borowy, 2013) shows how case studies can provide cues on the likely impacts (here health effects) of degrowth proposals.

Empirical assessments in the economic domain are scarce; attempts to quantify the increasing costs and disutility of continued economic growth are largely absent from the degrowth discourse. Behavioral aspects around instrumental conditioning, habits, and behavioral look-ins in favor of prevailing economic conditions have not been addressed in the context of degrowth. Complementary empirical research could also seek to understand the existing potentials for continued incremental growth that expands the economy asymptotically towards the planetary boundaries. The effects of zero interest and increasing reserve requirements for banking could be investigated further (as done, e.g., by Dittmer, 2015). Also in this respect, case studies such as those by Canavan (2013) on the declining tourism on the Isle of Man could examine macro- and micro-economic impacts of degrowth at a local scale and help addressing the hypothesis of Garcia (2012) according to which contraction may lead to greater economic diversifications.

#### 5.1.4 Engineering and Technological Innovation

Engineering and technological innovation have been dealt with only anecdotally in the degrowth discourse. The situation is about to change with a special issue forthcoming on this topic in fall 2016 (see Kerschner et al., 2015). The prevailing faith in technology appears to be deeply rooted in Western culture since Ancient Greek times. The theatrical representation of a comedy in which, when everything is devoted to a tragedy, the impossible occurs and saves the hero, was staged by the appearance of a *Deus ex machina*, both an image and a name that chiefly summarizes the dogmatic belief in progress through technology (Rist, 2002).

Degrowth supporters, instead, are more skeptical about technology that tends to ease access to natural resources, thereby expanding the resource base and thus the scale of the economy with negative consequences degrowth aims to mitigate in first place. However, one stream of thought also acknowledges the virtues of technology, for example as (i) a driver for increasing labor productivity (van den Bergh, 2011, Nørgård, 2013) which, in turn, might enable a reduction in working time and (ii) a means to decrease the negative environmental and health impacts of production and consumption. In fact, a versatile and innovative technology stock can enable factor substitution in face of environmental adversity. The rising sales and declining prices of hybrid cars after the oil price peak in 2008 (Weiss et al., 2012) or the emergence of electric two-wheelers in China in response to urban air pollution (Weiss et al., 2015) highlight the importance of technological innovation for maintaining well-being (here mobility) in times of increasing resource scarcity and adverse health impacts.

The often anecdotal treatment of engineering and technological innovation in the context of degrowth leaves space for more in-depth research. Kunze and Becker (2015) link renewable energy production to a political issue (namely collective ownership). We see ample grounds for similar analysis in the whole area of information technology and social media and their impact on production, consumption, and living patterns. Economic transitions could be assessed, such as shifts from low-value added towards high-value added products, from selling products to providing services, or from market transactions to non-market interactions, for example, within the sharing economy or as part of social enterprises (Johanisova and Wolf, 2012, Johanisova et al., 2013).

Largely unexplored remains the whole field around the effects of technology on society, specifically conviviality. The development of information technologies and social media as communication tools in their relation to degrowth is a fundamental issue to be analyzed: How can they catalyze a paradigm shift through democratic processes? To what extent can they: (i) contribute to degrowth and conviviality, (ii) facilitate a more decentralized society, or conversely (iii) punish deviant behavior and (iv) facilitate control of the masses? Finally, degrowth research itself could benefit from technology by employing increasingly powerful open-source IT tools for the analysis of, e.g., social media data.

#### 5.1.5 Implementation of Degrowth

The global cultural and institutional heritage suggests that degrowth transitions will likewise be diverse (Buch-Hansen, 2014), resulting in hybrid systems that comprise both elements of degrowth and the historically prevailing order (Boonstra and Joosse, 2013). While governance will arguably be critical to manage degrowth transitions (Borowy, 2013), the definition of verifiable targets might be equally important: By how much and until when will certain activities have to degrow so that persisting sustainability shortfalls can be addressed? The answer to such a question will likely be case-specific and subject to normative considerations. Yet, once clarified, implementation strategies for concrete policies could be devised.

Fotopoulos, 2010a, Fotopoulos, 2010b and Trainer, 2010, Trainer, 2014 emphasize the incompatibility of degrowth and the market economy but elaborate little on concrete policy scenarios to foster a large-scale transition towards more equitable societies. A reduction in working time (e.g., van den Bergh, 2011, Alcott, 2013, Knight et al., 2013, Andreoni and Galmarini, 2014, Fitzgerald et al., 2015) represents a concrete proposal that has received broader attention and could indeed find wide-spread support.

The implementation of dedicated policies may depend on the communication of degrowth objectives and practices to a wider audience. The importance of advertisement and nudging is touched by, e.g., van den Bergh (2011) and Spangenberg (2014); yet analyses on the effectiveness of information channels, including the aforementioned social media, to change consumer behavior and social norms are yet missing. Anecdotal evidence suggests that policies hampering business activity and consumption (van den Bergh, 2011) through taxation, cost internalization, environmental and labor standards without providing clear benefits to a larger group of stakeholders will face severe political opposition.

A limited number of case-studies has analyzed grass-root projects such as housing communities, eco-villages, rurban squats and concrete historical recessions cases (e.g., Cattaneo and Gavaldà, 2010, Lietaert, 2010, Nierling, 2012, Boillat et al., 2012, Canavan, 2013, Xue, 2014). As the findings of Xue (2014) suggest, spatial decentralization in eco-villages may come with negative social and environmental impacts. We see important knowledge gaps in the monitoring of existing degrowth implementations and the assessment of direct and indirect sustainability impacts, which can then lead to an adaptation of practices.

### 5.2 Hypotheses Towards a Research Program on Degrowth

Could degrowth receive a wider public support? Critics such as Tokic (2012), Romano (2012), or Schwartzman (2012), who consider degrowth an unfeasible elitist project would probably say no. We challenge this view with the following hypotheses:

• Degrowth could become popular once larger parts of the population have reached an affluence level beyond which the marginal utility of income, possibly quantified in terms of life satisfaction, becomes negative, that is, when economic growth at societal scale becomes uneconomic (see, e.g., Daly, 1999).
• Degrowth could become popular if its benefits are immediate and concrete both for the individual and the society and if experiences are widely shared, adapted, and discussed.
• Already the contemporary socio-economic system resembles features of a degrowth economy (examples comprise organic farming, the implementation of climate targets, Peer-to-Peer sharing). Mapping such features and understanding their principle drivers and obstacles can identify viable connection points for degrowth implementations.

Research on degrowth still occupies only a niche in the academic literature (Vandeventer, 2016). A diversified research program could (i) challenge the prevailing skepticism against degrowth by devising clear situations, contexts, and boundaries for which the dominant growth narrative proves wrong and (ii) shift the narrative away from ideas of adversity, recession or, at best, wishful thinking to focus on the benefits of degrowth for a prosperous and equitable human development.

But how could the predominantly normative character of degrowth be supported empirically, specifically if scientific reductionism may fail to portrait the width of the degrowth imaginary? To illustrate just part of the complexity: A set of functionally independent degrowth objectives *n* can lead to 2^*n*^ qualitatively distinct outcomes,^6^ spanning a huge space of explorable degrowth transitions. A well-articulated research program would need to bridge science, human nature, and the diversity of living conditions.

First, degrowth is heavily informed by the de-commodified, non-market sphere, comprising grass-root proposals, practices of voluntary simplicity, bottom-up initiatives, and network creating experiences. Many of these activities are grounded in economic anthropology and oppose the neoclassical paradigm of the *homo oeconomicus*. Research could map this non-market sphere, focusing on time-use allocation in specific non-monetary value-creating contexts and alternative economic practices (see e.g., the study of Conill et al. (2012) on Catalonia that could be extended to a larger set of countries). Research could seek to capture the variety of economic conditions or to dimension the potential of the Peer-to-Peer phenomenon (Kostakis et al., 2016). Such research could also include value-creating practices performed as part of household chores, the amateur economy (Nørgård, 2013), or unpaid work (D'alisa and Cattaneo, 2013).

In Polanian terms, research on the non-market sphere of value creation would quantify the embedded economy at multiple levels, ranging from the individual and its community to the city or country.^7^ Such an empirical research could help giving the non-market sphere visibility by quantifying (i) the time dedicated and benefits obtained from non-monetary practices and (ii) the relationships between market and non-market practices. In parallel, a proper dataset of alternative economic cultures should be created as a global atlas, locating in space and in context alternative practices.

Second, the issue of decreasing paid working time and expanding non-paid working time in the non-market sphere could be an avenue for empirical testing. Research could depart from cases in which work time reduction has occurred, such as in France (Haiden, 2006, Fagnani and Letablier, 2004) or was implemented in a particular context (such as in Sweden; TNYT, 2016) and expand with global surveys to understand the choices of citizens regarding their work-life balance and the socio-economic factors affecting such choices. Tests could devise (i) real effects of work time reduction on income, expenditure, leisure time, social interactions as well as (ii) hypothetical effects of a proposed work time reduction in the context of culture, gender, income, education, or geographical location to answer the question: What would citizens do with extra spare time?

Third, related to the lack of formal modelling identified in Section 5.1, equilibrium agent-based or system-dynamics models to be run with empirical data could address three topics: First, the issue of decoupling economic growth from CO_2_ emissions (and from other environmental impacts such as material use, land use change, or loss of biodiversity) could be modelled and empirically tested. For the past few decades, energy efficiency and the deployment of renewable energies have progressed at the global level too slowly (IEA, 2012) to adequately mitigate anthropogenic CO_2_ emissions. Examples of best practices from countries that have achieved a substantial reduction in CO_2_ emissions despite growing their GDP could be elaborated. The theoretical background would be the I = PAT equivalence (Jackson, 2009) applied at country level, over the past few decades, in which trade-related emissions are included (UNEP, 2016).

Likewise, modelling could be employed to predict the feasibility of decarbonizing GDP under different energy intensity scenarios; the scenarios could be based on past trends in which cases of astonishing technological improvement are documented.

Second, since the fear of unemployment, to a large extent driven by increasing productivity and a global division of labor, constitutes an important objection against degrowth, models that show how job creation can be decoupled from economic growth could be created. If properly calibrated, such models could be run on empirical macroeconomic data and predict trends and conditions in which such decoupling would occur. The models could also demonstrate how and under which conditions well-being effects materialize. Bilancini and D'Alessandro (2012) address this subject by investigating with an endogenous growth model (that accounts for the externalities of consumption, leisure, and production) the well-being effects of hypothetical degrowth transitions. Heikkinen (2015) has expanded this model which could be further advanced by linking leisure and non-market production to study well-being effects of specific policy interventions. Finally, the feedback loops identified by Videira et al. (2014) can provide the ground for modelling that seeks to parameterize variables, quantify the magnitude of feedbacks, and assess the impact of dedicated interventions in view of the various degrowth objectives.

## 6 Discussion

Degrowth is a marginal left-wing European position (Krugman, 2014) in the tradition of social and environmental activism. The degrowth discourse spans an increasingly diverse body of multi-disciplinary research at the intersection of social and environmental sciences.

With the methodological limitations sketched in Section 2, the outcome of our review suggests that the history, concept, and rationale for degrowth are well explained. Yet, the largely descriptive academic discourse lacks rigid hypotheses testing through modelling and empirical assessments. By addressing the research questions and hypotheses identified in Section 5, the academic degrowth discourse could make an important contribution to the debate around a sustainable post-growth development (see also Escobar, 2015).

We expect that degrowth may only receive broader public support if the marginal benefits of the status quo become smaller than those of the next best degrowth scenario for large parts of the population. The degrowth discourse has qualitatively discussed the deficiencies of the status quo but spent little effort to quantify the costs of continued economic growth as well as the well-being benefits of degrowth.

Moreover, growth policies may not necessarily be abandoned on a finite planet earth. Instead, such policies may allow making maximum use of available resources (be it through expanded resource extraction, technological innovation, or increased commodification of society) in the short term, while in parallel enabling the development of means to cope with environmental limits in the long term. Drought in California arguably forced residential water consumption to decrease in 2014 by some 30% (Reese, 2015) without causing major social disruptions. Such a decrease may not have been achievable by appealing to voluntary frugality nor may have water-saving policies obtained sufficient public support by pointing out unsustainable water consumption. The observed water savings might be temporary but show the capacity of humans to adapt in face of acute resource shortage. The case also points to the importance of technology as a catalyst for factor substitution in production and consumption in response to environmental constraints.

To be successful, degrowth has to identify a concrete and inclusive development perspective (see Schwartzman, 2012) for the affluent and powerful elites and the marginalized poor. Direct benefits of degrowth might be experienced by consumers in areas where further growth has obviously become undesirable, such as in the health care industry as illustrated by Missoni (2015), in the food, nutrition and the agricultural sector, or in urban transportation. Degrowth could address psychological stress related to over consumption, long working hours, and the commodification of social relations and highlight the benefits of a simplified life style away from positional competition and towards more collaborative community development. Addressing life quality around resonant human interactions (Rosa, 2015) in face of increasing competition and individuation may be a viable angle to highlight the benefits of degrowth. Decreasing working time can mitigate environmental degradation (Knight et al., 2013, Fitzgerald et al., 2015) and provide a leverage point for virtually all other degrowth proposals. In fact, we would regard a decrease in working time as the single silver bullet through which degrowth can yield personal welfare gains, increase environmental sustainability, enhance democracy, and thus obtain the support of larger parts of the population. Yet, to be a fulfilling choice, reduced working time, and degrowth in more general, may hinge on a wider cultural recognition (see, e.g., Skidelsky and Skidelsky, 2012) that still appears to be hampered under the present societal conditions.

Kallis (2013) argues that societies have the capacity to steer social processes towards degrowth, thereby opposing the view of Sorman and Giampietro (2013) who consider that societies are destined to grow, crash, and adapt. We see a larger and more differentiated space of development to which the degrowth discourse contributes visions for both social and economic adaptation and the mitigation of environmental impacts. In a resource-constraint world, degrowth may occur as a gradual and locally-specific transition (Buch-Hansen, 2014). We argue with Ott (2012) in favor of political prudence through addressing specific problems with specific policies and against the pursuit of grand new utopias that often come with unintended consequences.

## 7 Conclusions

Throughout millennia, the anthroposphere has been expanding both as a consequence and driver of human development. On a finite planet, this expansion will eventually come to an end. The political degrowth movement has established a discourse about the fundamental questions around this perspective. Whether the limits to growth should be anticipated by policy (as proposed by degrowth advocates) or dealt with retrospectively in face of acute adversity, is a normative rather than scientific question. Yet, the academic degrowth discourse can help testing hypotheses, provide models, empirical data, and an alternative vision of human development. Degrowth may reach a wider audience if it can identify and communicate concrete well-being benefits. If degrowth is a political slogan (Latouche, 2010) with theoretical and practical implications, the academia has just began to analyze these. By developing positive visions, and presenting implementable solutions, degrowth could contribute to a prosperous yet equitable, participatory, and environmentally sustainable society.

## Disclaimer and Acknowledgements

The views expressed here are those of the authors and should not be regarded as the official position of the European Commission. We thank Blake Alcott, Samuel Alexander, Valeria Andreoni, Viviana Asara, Christine Bauhardt, Barış Gençer Baykan, Ennio Bilancini, Mauro Bonaiuti, Wijnand Boonstra, Iris Borowy, Leigh Brownhill, Hubert Buch-Hansen, Brendan Canavan, David Correia, Roberto De Vogli, Federico Demaria, Laia Domènech, Salvatore Engel-Di Mauro, Andreas Exner, Takis Fotopoulos, Valerie Fournier, Ernest Garcia, Julien-François Gerber, Juan Infante-Amate, Naděžda Johanisová, Giacomo D'Alisa, Panagiotis Kalimeris, Giorgos Kallis, Christian Kerschner, Kent A. Klitgaard, Sylvia Lorek, Volker Mauerhofer, Barbara Muraca, Linda Nierling, Jørgen Nørgård, Konrad Ott, Salvador Pueyo, François Schneider, David Schwartzman, Alevgul Sorman, Joachim Spangenberg, Frederick Trainer, Mark Whitehead, Jeroen van den Bergh, Pascal van Griethuysen, Peter Victor, Nuno Videira, and Jin Xue for supporting the review of the degrowth literature. We are grateful for the help of Juliana Stropp on Fig. 3, the stimulating discussions with Bart Degraeuwe, and the comments of two anonymous reviewers on an earlier draft of this article.

The following is the supplementary data related to this article.

Supplemental Table S1 Supplemental Table S1

## Figures and Tables

**Table A1:** Key features of the reviewed articles; assertions presented here reflect our understanding of the respective article but may not capture in all detail the message intended by the authors

**History and context - Degrowth:** -entered French politics in 1979 (1), echoes the utopist ideas of Rousseau and Godwin (6), draws from the theoretical contributions of Gorz (54), and merges ideas of Francophone décroissance and Anglophone ecological economics (8) -was subject of disagreement between Georgescu-Roegen and the Club of Rome (7) and united in 2007 French and Italian proponents of convivial, autonomous, and sustainable societies (3) -entered the academic debate after the first degrowth conference (Paris, France, 2008; 56) **Characterization of the economy** -capitalist and socialist economies are growth oriented and intrinsically unsustainable (2) - globalization broke the historical balance between capitalism and democracy (42) and triggered a spiral of environmental, economic, and social crises (23) -property expansion subordinates society and environment under capitalist requirements (15) and leads to power concentration, circular self-expansion, path-dependent lock-in, discouraging non-compliant behavior (36) -increasing energy prices absorb capital and enhance the risk of debt default and economic recession (48) -negative growth-related impacts are relevant in high-income Eastern regions of China (38) **Elements of systems theory** - a systems perspective can reveal mechanisms underlying the market economy (32) that constitutes a complex system, operating between order and chaos, displaying scale invariance, power-law distributions, phase transitions, and self-organized criticality (79)	**Conceptual aspects – Degrowth:** -is a **political** slogan with theoretical implications, revolving around 8 R’s: revalue, re-conceptualize, restructure, relocate, redistribute, reduce, reuse, and recycle (12) -constitutes a **paradigmatic proposal** for an equitable downscaling, revitalized democracy, and improved environmental conditions (14,22,84) that criticizes sustainable development and argues in favor of anti-utilitarianism, bio-economics, justice (52), and inclusive democracy through the establishment of a popular base of political and economic power (16) -calls for a new social, utopic, and subversive imaginary (82), reclaimed democracy and re-politicized economic relations and identities (4) - embraces cultural and political change (20) whose relationship to democracy can be clarified by Castoriadis’ thoughts on democracy and autonomy (50) -means a **voluntary** transition towards a just, participatory, and ecologically sustainable society (9) through a bottom-up process that shares views of the political left and right in France (1) -requires management (68) and could be shaped by social practices implemented by socio-environmental movements (41) and the goal of a steady-state economy (11,51) -trajectories include demonetized, self-organized forms of commons-based solidarity economies (70) -is inevitable as fossil fuels become scarce (30) and the Northern countries path towards a steady-state economy (10); transition will take different forms and lead to a variety of outcomes due to institutional and cultural diversity (76) -calls for a reduction of paid but not **un-paid work** and may not *per se* increase institutional costs (63): a decrease in production and consumption and an increase in leisure appears feasible, can be socially sustainable (29) and increase overall welfare (89) -is subject to understanding the functional lock-in of the economy and subordinating the latter to social and ecological imperatives (15) -can draw from the insight gained by **ecological** economics (31), responds to the physical, ecological, social, and economic limits and draws from a vibrant research agenda (66) -can be assessed by applying the concept of environmental space (78) and biophysical and social indicators based on a unifying conceptual framework (34) -alters societal metabolism, human life styles (67), and is linked to strong sustainable consumption through various interdependencies (64) -interventions, feedback, and leverage points can be understood through causal loop diagrams (73) and could be normatively underpinned by Sen’s and Nussbaum’s capabilities approach (44) -necessitates a fundamental transformation of institutions (23), can lead to further democratization on grounds of Habermas’ deliberative democracy (45) and be pursued through environmental and redistributive policies (20), fostering social protection and income equality (80) -potentials for conscious change can be identified by taking an evolutionary perspective (43) -will show bricolage, translation, path-dependency, and inherited legacy of diverse cultural conditions (76), may be associated with increased competition and regulatory interventions (53), could be be supported by payments for environmental services as a transition instrument (90)	**Complementary initiatives** -post-development, sharing with degrowth common imaginaries, goals, and predicaments (85) -environmental justice movement (5,26), inclusive democracy (16,17), and transition movement (55) -ecofeminist economics (74), earth jurisprudence (19), and de-alienation to re-integrate humans in harmony with nature (24) -bourgeois primitivism that propagates environmentalism as self-improvement exercise for the elite (25) **Deviant views and degrowth critique** -Latouche and Fotopoulos hold misconceptions about Castoriadis’ notion of autonomy, which limits their view on degrowth trajectories (50) -degrowth is ambiguous, ineffective, and unfeasible – a-growth should be pursued by effective sustainability policies (21) -degrowth is inadequate to address the multidimensional crisis (17) – The ‘Simpler Way’ constitutes a concrete eco-anarchist vision for local self-sustained communities (71) -degrowth does not provide a concrete positive vision of the future (27) but would cause the economy to implode and eventually grow again (35) -degrowth insufficiently addresses militarism, imperialism, and the observation that the various forms of growth differ in their environmental and social effects (28) -degrowth-democracy relationship is problematic (46) **Empirical insights** **-**public support for degrowth is small but higher in wealthy than in poor European countries (83) -health trends are inconsistent during the economic crisis in the 1990s in Cuba (58) and in 2008 in northern countries (80) -declining tourism on the Isle of Man (UK) caused economic diversification and negative environmental and social impacts (68) **-**degrowth could decrease Canadian GHG emissions by 80% between 2005 and 2035 (37) -localization and decentralization by eco/urban villages can contradict environmental degrowth objectives (75) -half (550 Mt) of the global copper reserves are extracted; a global per capita copper stock of 100 kg appears to be sustainable (69) -empirical data inconclusive about causality between energy use and GDP (77) -the Spanish agri-food system consumes 1408 PJ primary energy, which is 7.4 times the residential energy use in Spain (61) -the energy intensity of unpaid work in Catalonia is lower than that of the service and governmental sector (59) -growth in housing stock has only weakly decoupled from environmental impacts (91) **Degrowth implementations through:** -cohousing (13) and rurban squats (10), voluntary frugality, and a simpler way of living (39,47,49) -cooperative, self-governed, and local economies (18) -non-market capitals exchanged by primary and secondary social enterprises (62) -reduction of paid work, expansion of reciprocity work and unpaid work (33,72) in an amateur economy (72) and a universal job guarantee that decouples the labor market from economic development (57) -autonomous small farmer’s cooperatives (40) and co-production of farmers and consumers via community-supported agriculture (81) -manufacturing on high-value added niche products such as gluten-free beer (88) -reuse of reclaimed water and desalination (60), collective ownership of renewable energy supply projects (86) -reformed global health governance and degrowth in the health care industry (87)

**Fig. 6:**
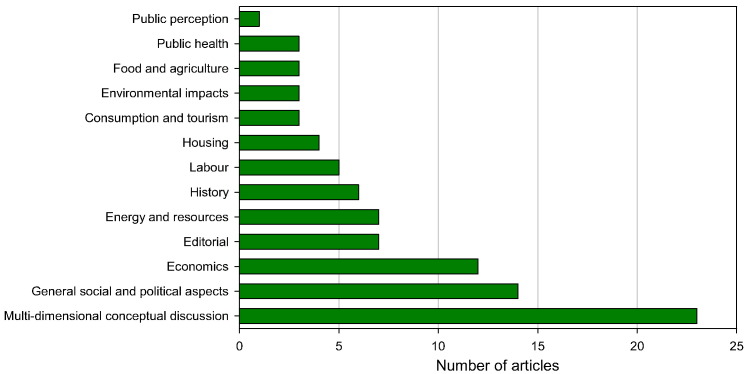
Thematic overview of the academic degrowth discourse based on 91 peer-refereed articles published between 2007 and 2015.

## Footnotes

1 We deviate from this approach in the case of Muraca (2013) who refers in the title of her article to ‘Décroissance’, i.e., the French synonym for ‘degrowth’.

2 The number of relevant web pages is identified by using quotation marks as Google search operator.

3 The historic observation that decoupling between resource use and gross domestic product did not happen at global scale does not necessarily imply that decoupling under constrained resource availability is absolutely infeasible (see however Ward et al. (2016), who challenge this assertion).

4 Brownhill et al. (2012) argue in the same direction when asking whether degrowth could present a business plan for enterprises.

5 Van den Bergh (2011) and Schwartzman (2012) doubt that voluntary degrowth finds sufficient public support, while a more recent survey by Drews and van den Bergh (2016) suggests that a third of Spanish citizens support degrowth as a policy objective.

6 We assume here that pursuing an objective can lead to an increase or decrease of a given parameter. If one assumes that pursuing an objective could also leave the parameter unchanged, the space of stylized degrowth transitions increases to 3^*n*^.

7 As for neoclassical economics, postulates could be posed based on realistic anthropological cases and a new economic theory (i.e., re-embedded economics), which then could be tested with econometrics. Furthermore, linking environmental history with the analysis of the social metabolism of agrarian societies could help to interpret the relationship between societies that accumulate and societies, deeply investigated in anthropology, that do not accumulate and are more egalitarian.
