# Supplementary material for: Degrowth – Taking Stock and Reviewing an Emerging Academic Paradigm
Source: Ecol Econ. 2017 Jul;137:220–30. doi: 10.1016/j.ecolecon.2017.01.014 (PMC5421156; doi:10.1016/j.ecolecon.2017.01.014)
Supplement: Supplemental Table S1 [file mmc1.docx]

**Supplementary Material**

Table S1: Overview - peer-refereed articles published before 31 December 2015 with the word ‘degrowth’ or ‘de-growth’ in the title; articles are ordered according to (i) year of publication, (ii) journal name, and (iii) author name

| Number | Year | Authors | Title | Main research topic related to degrowth | Journal |
| --- | --- | --- | --- | --- | --- |
| 1 | 2007 | Baykan | From limits to growth to degrowth within French green politics | History | Environmental Politics 16 |
| 2 | 2007 | Fotopoulos | Is degrowth compatible with a market economy? | Economics | The International Journal of Inclusive Democracy 3 |
| 3 | 2007 | Latouche | De-growth: An electoral stake? | General social and political aspects | The International Journal of Inclusive Democracy 3 |
| 4 | 2008 | Fournier | Escaping from the economy: The politics of degrowth | History | International Journal of Sociology and Social Policy 28 |
| 5 | 2009 | Martínez-Alier | Socially sustainable economic de-growth | Environmental impacts | Development and Change 40 |
| 6 | 2009 | Sippel | Back to the future: Today’s and tomorrow’s politics of degrowth economics (décroissance) in light of the debate over luxury among eighteenth and early nineteenth century utopists | History | International Labor and Workings-Class History 75 |
| 7 | 2010 | Levallois | Can de-growth be considered a policy option? A historical note on Nicholas Georgescu-Roegen and the Club of Rome | History | Ecological Economics 69 |
| 8 | 2010 | Martínez-Alier et al. | Sustainable de-growth: Mapping the context, criticisms and future prospects of an emergent paradigm | Multi-dimensional conceptual discussion | Ecological Economics 69 |
| 9 | 2010 | R&D^1^ | Degrowth declaration of the Paris 2008 conference | Multi-dimensional conceptual discussion | Journal of Cleaner Production 18 |
| 10 | 2010 | Cattaneo and  Gavaldà^1^ | The experience of rurban squats in Collserola, Barcelona: What kind of degrowth? | Housing | Journal of Cleaner Production 18 |
| 11 | 2010 | Kerschner^1^ | Economic de-growth vs. steady-state economy | Multi-dimensional conceptual discussion | Journal of Cleaner Production 18 |
| 12 | 2010 | Latouche^1^ | Editorial – Degrowth | History | Journal of Cleaner Production 18 |
| 13 | 2010 | Lietaert^1^ | Cohousing’s relevance to degrowth theories | Housing | Journal of Cleaner Production 18 |
| 14 | 2010 | Schneider et al.^1^ | Crisis or opportunity? Economic degrowth for social equity and ecological sustainability | Editorial | Journal of Cleaner Production 18 |
| 15 | 2010 | van Griethuysen^1^ | Why are we growth-addicted? The hard way towards degrowth in the involuntary western development path | Economics | Journal of Cleaner Production 18 |
| 16 | 2010a | Fotopoulos | Direct Democracy and de-growth | General social and political aspects | The International Journal of Inclusive Democracy 6 |
| 17 | 2010b | Fotopoulos | De-growth, the Simpler Way and Inclusive Democracy | General social and political aspects | The International Journal of Inclusive Democracy 6 |
| 18 | 2010 | Trainer | De-growth – is not enough | Multi-dimensional conceptual discussion | The International Journal of Inclusive Democracy 6 |
| 19 | 2010 | Alexander | Earth jurisprudence and the ecological case for degrowth | Multi-dimensional conceptual discussion | The Journal of Jurisprudence 6 |
| 20 | 2011 | Kallis | In defence of degrowth | Multi-dimensional conceptual discussion | Ecological Economics 70 |
| 21 | 2011 | van den Bergh | Environment versus growth – A criticism of “degrowth” and a plea for “a-growth” | Multi-dimensional conceptual discussion | Ecological Economics 70 |
| 22 | 2011 | Schneider et al. | Sustainable degrowth | Multi-dimensional conceptual discussion | Journal of Industrial Ecology 15 |
| 23 | 2012 | Bonaiuti^2^ | Degrowth: Tools for a complex analysis of the multidimensional crisis | Multi-dimensional conceptual discussion | Capitalism Nature Socialism 23 |
| 24 | 2012 | Brownhill et al. ^2^ | Degrowth? How about some “De-alienation”? | General social and political aspects | Capitalism Nature Socialism 23 |
| 25 | 2012 | Correia^2^ | Degrowth, American style: No impact man and bourgeois primitivism | Multi-dimensional conceptual discussion | Capitalism Nature Socialism 23 |
| 26 | 2012 | Martínez-Alier^2^ | Environmental justice and economic degrowth: An alliance between two movements | General social and political aspects | Capitalism Nature Socialism 23 |
| 27 | 2012 | Schwartzman^2^ | A critique of degrowth and its politics | Multi-dimensional conceptual discussion | Capitalism Nature Socialism 23 |
| 28 | 2012 | Saed^2^ | Introduction to the degrowth symposium | Editorial | Capitalism Nature Socialism 23 |
| 29 | 2012 | Bilancini and D’Alessandro^3^ | Long-run welfare under externalities in consumption, leisure, and production: A case for happy degrowth vs. unhappy growth | Economics | Ecological Economics 84 |
| 30 | 2012 | Douthwaite^3^ | Degrowth and the supply of money in an energy-scarce world | Economics | Ecological Economics 84 |
| 31 | 2012 | Kallis et al.^3^ | The economics of degrowth | Editorial | Ecological Economics 84 |
| 32 | 2012 | Klitgaard and  Krall | Ecological economics, degrowth, and institutional change | Economics | Ecological Economics 84 |
| 33 | 2012 | Nierling^3^ | “This is a bit of the good life”: Recognition of unpaid work from the perspective of degrowth | Work | Ecological Economics 84 |
| 34 | 2012 | O’Neill^3^ | Measuring progress in the degrowth transition to a steady state economy | Multi-dimensional conceptual discussion | Ecological Economics 84 |
| 35 | 2012 | Tokic^3^ | The economic and financial dimensions of degrowth | Economics | Ecological Economics 84 |
| 36 | 2012 | van Griethuysen^3^ | Bona diagnosis, bona curatio: How propperty economics clarifies the degrowth debate | Economics | Ecological Economics 84 |
| 37 | 2012 | Victor^3^ | Growth, degrowth and climate change: A scenario analysis | Environmental impacts | Ecological Economics 84 |
| 38 | 2012 | Xue et al. | Is the degrowth debate relevant to China? | Economics | Environment, Development and Sustainability 14 |
| 39 | 2012a | Alexander | Planned economic contraction: The emerging case for degrowth | Economics | Environmental Politics 21 |
| 40 | 2012 | Boillat et al.^4^ | What economic democracy for degrowth? Some comments on the contribution of socialist models and Cuban agroecology | Food and agriculture | Futures 44 |
| 41 | 2012 | Cattaneo et al.^4^ | Introduction - Degrowth futures and democracy | Editorial | Futures 44 |
| 42 | 2012 | Deriu^4^ | Democracies with a future: Degrowth and the democratic tradition | General social and political aspects | Futures 44 |
| 43 | 2012 | Garcia^4^ | Degrowth, the past, the future, and the human nature | General social and political aspects | Futures 44 |
| 44 | 2012 | Muraca^4^ | Towards a fair degrowth-society: Justice and the right to a ‘good life’ beyond growth | General social and political aspects | Futures 44 |
| 45 | 2012 | Ott^4^ | Variants of de-growth and deliberative democracy: A Habermasian proposal | General social and political aspects | Futures 44 |
| 46 | 2012 | Romano^4^ | How to rebuild democracy, re-thinking degrowth | General social and political aspects | Futures 44 |
| 47 | 2012 | Trainer^4^ | De-growth: Do you realize what it means? | Multi-dimensional conceptual discussion | Futures 44 |
| 48 | 2012b | Alexander | Degrowth, expensive oil, and the new economics of energy | Energy and resources | Real-World Economics Review 61 |
| 49 | 2013a | Alexander^5^ | Voluntary simplicity and the social reconstruction of law: Degrowth from the grassroots up | General social and political aspects | Environmental Values 22 |
| 50 | 2013 | Asara et al. ^5^ | Degrowth, democracy and autonomy | General social and political aspects | Environmental Values 22 |
| 51 | 2013 | Boonstra and  Joosse^5^ | The social dynamics of degrowth | Multi-dimensional conceptual discussion | Environmental Values 22 |
| 52 | 2013 | Demaria  et al. ^5^ | What is degrowth? From an activist slogan to a social movement | Multi-dimensional conceptual discussion | Environmental Values 22 |
| 53 | 2013a | Mauerhofer^5^ | Lose less instead of win more: The failure of decoupling and perspectives for competition in a degrowth economy | Multi-dimensional conceptual discussion | Environmental Values 22 |
| 54 | 2013 | Muraca^5^ | Décroissance: A project for a radical transformation of society | History | Environmental Values 22 |
| 55 | 2013 | Quilley^5^ | De-growth is not a liberal agenda: Relocalisation and the limits to low energy cosmopolitanism | Multi-dimensional conceptual discussion | Environmental Values 22 |
| 56 | 2013 | Whitehead^5^ | Degrowth or regrowth? | Editorial | Environmental Values 22 |
| 57 | 2013 | Alcott^6^ | Should degrowth embrace the Job Guarantee? | Work | Journal of Cleaner Production 38 |
| 58 | 2013 | Borowy^6^ | Degrowth and public health in Cuba: Lessons from the past? | Public health | Journal of Cleaner Production 38 |
| 59 | 2013 | D’Alisa and  Cattaneo^6^ | Household work and energy consumption: A degrowth perspective. Catalonia’s case study | Work | Journal of Cleaner Production 38 |
| 60 | 2013 | Domènech et al. ^6^ | Degrowth initiatives in the urban water sector? A social multi-criteria evaluation of non-conventional water alternatives in Metropolitan Barcelona | Energy and resources | Journal of Cleaner Production 38 |
| 61 | 2013 | Infante-Amate and González de Molina^6^ | ‘Sustainable de-growth’ in agriculture and food: an agro-ecological perspective on Spain’s agri-food system (year 2000) | Energy and resources | Journal of Cleaner Production 38 |
| 62 | 2013 | Johanisova et al. ^6^ | Social enterprises and non-market capitals: a path to degrowth? | Economics | Journal of Cleaner Production 38 |
| 63 | 2013 | Kallis^6^ | Societal metabolism, working hours and degrowth: A comment on Sorman and Giampietro | Multi-dimensional conceptual discussion | Journal of Cleaner Production 38 |
| 64 | 2013 | Lorek and  Fuchs^6^ | Strong sustainability consumption governance – precondition for a degrowth path? | Consumption and tourism | Journal of Cleaner Production 38 |
| 65 | 2013 | Nørgård^6^ | Happy degrowth through more amateur economy | Work | Journal of Cleaner Production 38 |
| 66 | 2013 | Sekulova et al.^6^ | Degrowth: From theory to practice | Editorial | Journal of Cleaner Production 38 |
| 67 | 2013 | Sorman and Giampietro^6^ | The energetic metabolism of societies and the degrowth paradigm: analyzing biophysical constrains and realities | Energy and resources | Journal of Cleaner Production 38 |
| 68 | 2013 | Canavan | Sustainable tourism: Development, decline and de-growth. Management issues from the Isle of Man | Consumption and tourism | Journal of Sustainable Tourism 22 |
| 69 | 2014 | Exner et al. | Sold futures? The global availability of metals and economic growth at the peripheries: Distribution and regulation in a degrowth perspective | Energy and resources | Antipode 47 |
| 70 | 2014 | Exner | Degrowth and democratization: On the limits of a non-capitalist market economy | Multi-dimensional conceptual discussion | Capitalism Nature Socialism 25 |
| 71 | 2014 | Trainer | The degrowth movement from the perspective of The Simpler Way | Multi-dimensional conceptual discussion | Capitalism Nature Socialism |
| 72 | 2014 | Andreoni and Galmarini | How to increase well-being in a context of degrowth | Work | Futures 55 |
| 73 | 2014 | Videira et al. | Improving understanding on degrowth pathways: An exploratory study using collaborative causal models | Multi-dimensional conceptual discussion | Futures 55 |
| 74 | 2014 | Bauhardt | Solutions to the crisis? The green new deal, degrowth, and the solidarity economy: Alternatives to the capitalist growth economy from an ecofeminist economics perspective | General social and political aspects | Ecological Economics 102 |
| 75 | 2014 | Xue | Is eco-village/urban village the future of a degrowth society? An urban planner's perspective | Housing | Ecological Economics 105 |
| 76 | 2014 | Buch-Hansen | Capitalist diversity and de-growth trajectories to steady-state economies | Economics | Ecological Economics 106 |
| 77 | 2014 | Kalimeris et al. | A meta-analysis investigation of the direction of the energy-GDP causal relationship: implications for the growth-degrowth dialogue | Energy and resources | Journal of Cleaner Production 67 |
| 78 | 2014 | Spangenberg | Institutional change for strong sustainable consumption: Sustainable consumption and the degrowth economy | Consumption and tourism | Sustainability: Science, Practice, & Policy 10 |
| 79 | 2014 | Pueyo | Ecological econophysics for degrowth | Multi-dimensional conceptual discussion | Sustainability 6 |
| 80 | 2015 | De Vogli and  Owusu | The causes and health effects of the Great Recession: From neoliberalism to ‘healthy de-growth’ | Public health | Critical Public Health 25 |
| 81 | 2015 | Bloemmen et al. | Microeconomic degrowth: The case of community supported agriculture | Food and agriculture | Ecological Economics 112 |
| 82 | 2015 | Kallis and  March | Imaginaries of hope: The Utopianism of Degrowth | Multi-dimensional conceptual discussion | Annals of the Association of American Geographers 105 |
| 83 | 2015 | Ančić and  Domazet | Potential for degrowth: Attitudes and behaviours across 18 European countries | Public perception | Teorija in Praksa 52 |
| 84 | 2015 | Asara et al. ^7^ | Socially sustainable degrowth as a social-ecological transformation: Repoliticizing sustainability | Editorial | Sustainability Science 10 |
| 85 | 2015 | Escobar^7^ | Degrowth, postdevelopment, and transitions: A preliminary conversation | General social and political aspects | Sustainability Science 10 |
| 86 | 2015 | Kunze and  Becker^7^ | Collective ownership in renewable energy and opportunities for sustainable degrowth | Energy and resources | Sustainability Science 10 |
| 87 | 2015 | Missoni^7^ | Degrowth and health: Local action should be linked to global policies and governance for health | Public health | Sustainability Science 10 |
| 88 | 2015 | Harasym and Podeszwa | Towards sustainable de-growth – medical survey data as predictors for estimation of niche market value – gluten free beer market case | Food and agriculture | Journal of Cleaner Production 108 |
| 89 | 2015 | Heikkinen | (De)growth and welfare in an equilibrium model with heterogeneous consumers | Economics | Ecological Economics 116 |
| 90 | 2015 | Muniz and  Cruz | Making nature valuable, not profitable: Are payments for ecosystem services suitable for degrowth? | Environmental impacts | Sustainability 7 |
| 91 | 2015 | Xue | Sustainable housing development: Decoupling or degrowth? A comparative study of Copenhagen and Hangzhou | Housing | Environment and Planning C: Government and Policy 33 |

^1^Special issue on degrowth in the *Journal of Cleaner Production*; contribution originates from the first degrowth conference (Paris, France, 2008).

^2^Special issue on degrowth in the journal *Capitalism Nature Socialism*.

^3^Special issue on degrowth in the journal *Ecological Economics*; contribution originates from the second degrowth conference (Barcelona, Spain, 2010).

^4^Special issue on degrowth in the journal *Futures*; contribution originates from the second degrowth conference (Barcelona, Spain, 2010).

^5^Special issue on degrowth in *Environmental Values*.

^6^Special issue on degrowth in the *Journal of Cleaner Production*; contribution originates from the second degrowth conference (Barcelona, Spain, 2010).

^7^Special issue on degrowth in *Sustainability Sciences*; contribution originates from the third or fourth degrowth conferences (Venice, Italy, 2012; Leipzig, Germany, 2014).
